# Early HIV-1 Gag Assembly on Lipid Membrane with vRNA

**DOI:** 10.21203/rs.3.rs-3060076/v1

**Published:** 2023-07-03

**Authors:** Anne X.-Z. Zhou, John A. Hammond, Kai Sheng, David P. Millar, James R. Williamson

**Affiliations:** 1 Department of Integrative Structural and Computational Biology, The Scripps Research Institute; La Jolla, CA 92037, USA.

## Abstract

HIV-1 capsid assembly is an essential process in the virus infection cycle. Initiation of capsid assembly involves viral proteins, genomic RNA, and the inner leaflet of the plasma membrane, facilitated by a number of cellular factors^1^. The viral structural protein Gag plays a number of central roles in this process, including association with the membrane, selective binding of genomic RNA, and oligomerization and packaging to ultimately produce an immature budded pro-viral particle^2^. While there have been intensive studies regarding the early stages of Gag assembly, there is a lack of consensus on the mechanism for nucleation and growth of Gag complexes^3–7^. Here we show that myristoylated Gag forms a trimer nucleus in a model membrane that can selectively bind a dimeric RNA containing the packaging signal. Subsequent growth of myristoyl-Gag oligomers requires vRNA, and occurs by addition of 1 or 2 Gag monomers at a time from solution. These data support a model where the immature capsid lattice formation occurs by a gradual lattice edge expansion, following a trimeric nucleation event. The dynamic single molecule data that support this model were recorded using mass photometry, involving full length myristoylated protein, RNA, and lipid together. These data are the first to support a lattice edge expansion model of Gag during early stages of assembly in a biological-relevant setting, providing insights to the fundamental models of virus structural protein assembly process.

The global impact of acquired immunodeficiency syndrome (AIDS) has made human immunodeficiency virus (HIV) one of the most intensely studied viruses. To infect new cells, HIV must assemble infectious viral particles within the infected host. This assembly process is complicated and carefully choreographed among viral proteins, virus genomic RNA (gRNA), and the inner leaflet of the plasma membrane, involving many cellular host proteins^1^. The viral structural polyprotein Gag, accounting for more than half of the virus biomass, plays a crucial role in this process, with involvement of four domains that have different but coordinated functions: the matrix (MA) domain mediates binding to the membrane and specifically interacts with phosphatidylinositol 4,5-biphosphate (PI(4,5)P2) on the membrane, the capsid (CA) domain mediates association with other Gag proteins, the nucleocapsid (NC) domain confers selective binding to gRNA, and the p6 domain is responsible for recruiting cellular host factors^2^. The general functions of Gag are to oligomerize, selectively bind dimerized viral gRNA, and interact with the inner leaflet of the plasma membrane to form newly assembled immature virus particles.

Although virus assembly has been studied extensively over the years^3–7^, there are only a few biochemical studies involving the three key components: myristoylated full-length Gag, viral RNA, and lipid membranes. Recent studies using two of these three components have pointed to the intertwined interactions between Gag, gRNA, and lipids. Recent structural and biochemical results have identified a gRNA dimer that can facilitate Gag-RNA nucleation^8^, while there is mounting evidence showing that Gag-Gag interactions, via both CA domain and MA domain, are the main driving forces for the final immature lattice formation^9, 10^. The Gag CA domain forms stable hexamers in the immature lattice, and the Gag MA domain forms a hexamer of trimers on a model membrane system^11–13^. The two domains are connected by a flexible linker, possibly allowing both domains to adopt their preferred oligomerization pattern simultaneously^11, 13^. The interactions between MA domain and the lipid membrane add yet another layer of complexity to the assembly process, and there is no consensus on the mechanism for nucleation and growth of oligomeric Gag complexes. Thus, it is essential to investigate this important viral assembly process with all three components present in the system.

To monitor engagement of Gag on the membrane with gRNA in a single molecule approach, we harnessed the power of the newly developed technique of mass photometry. Mass photometry is based on principles of interferometric scattering (iSCAT) microscopy, where light is scattered by single macro-molecules near a slide surface can be detected in a label-free manner, and transformed into a contrast value by applying a model point spread function (PSF) to the resulting interferomic signal. This signal can then be converted to molecular weight using a mass standard calibration^14, 15^. The correlation between the amount of light scattered by a particle and the molecular weight of a particle is linear throughout a wide range, regardless of shape. Mass photometry data can be acquired in two observation modes. In a conventional mass photometry assay (landing assay), interference signals of individual molecules are detected as they adsorb nonspecifically on a slide surface. A histogram is created by observing a sufficiently large number of adsorption events, and the molecular weights of species present in solution can be observed in a histogram form. A second observation mode, called a mass-sensitive particle tracking (MSPT) assay, tracks individual molecules associating with and diffusing across a surface. In this mode, the changes in contrast patterns along a positional trajectory are recorded, allowing both positional dynamics and changes in the molecular weight to be observed directly at the same time (Fig. 1, S1)^16, 17^.

Using mass photometry, we examined the early steps in Gag assembly using myristoylated full-length Gag (myr-Gag), 5’ UTR gRNA containing the packaging signal^18^, and a supported lipid bilayer (SLB) containing PI(4,5)P2. We demonstrated the importance of the presence of lipid and gRNA in Gag oligomerization and showed that proper CA-mediated Gag dimerization is needed for assembly and efficient recruitment of gRNA to the membrane. Our data indicate that a Gag trimer may be the smallest nucleating unit on the membrane, that trimeric Gag associates with dimeric vRNA as a prerequisite for further Gag oligomerization in the membrane, and that the assembly proceeds via an edge-expansion process, where one or two Gag proteins are added at a time from the solution to the growing assembly nucleus.

## Myr-Gag primarily consists of monomers and dimers in solution

MP landing assays were used to monitor the oligomerization distribution of myr-Gag or myr-Gag-RNA complexes in binding buffer. Over the concentration range tested (10–100 nM), myr-Gag mainly consisted of monomeric and dimeric Gag populations (~55 and 110 kDa respectively) (Fig. S2C) When WT 5’ UTR RNA (352 nt, 110 kDa) was incubated with 50 nM myr-Gag, an additional small peak was observed, corresponding to an RNA dimer (220 kDa) (Fig. S2D). The 5’-UTR RNA used here is well known to form a dimeric species^19, 20^, which was confirmed using non-denaturing gel electrophoresis (Fig. S2A, S2B). Although a few particles with higher molecular weights were detected, the distribution of lower masses suggests that Gag-RNA complexes are not the major species present in solution (Fig. S2D). Neither myr-Gag alone nor myr-Gag with RNA form higher order complexes to any significant extent in solution at the concentrations tested here.

## HIV-1 5’ UTR RNA promotes further Gag assembly on SLBs

MSPT experiments were employed to track myr-Gag diffusion on a lipid membrane. Complexes diffusing in the SLB were readily observed and tracking of individual complexes yielded a substantial number of analyzable trajectories (Fig. S1). The myr-Gag concentration and the PI(4,5)P2 composition in the SLB were surveyed to identify 50 nM myr-Gag and 2% PI(4,5)P2 as a condition with a trajectory density that is sufficient to generate statistics within the field of view (FOV) without crowding or overlap, optimizing the quantity and quality of the data that can be extracted (Fig. S1E).

**Fig. 2.**
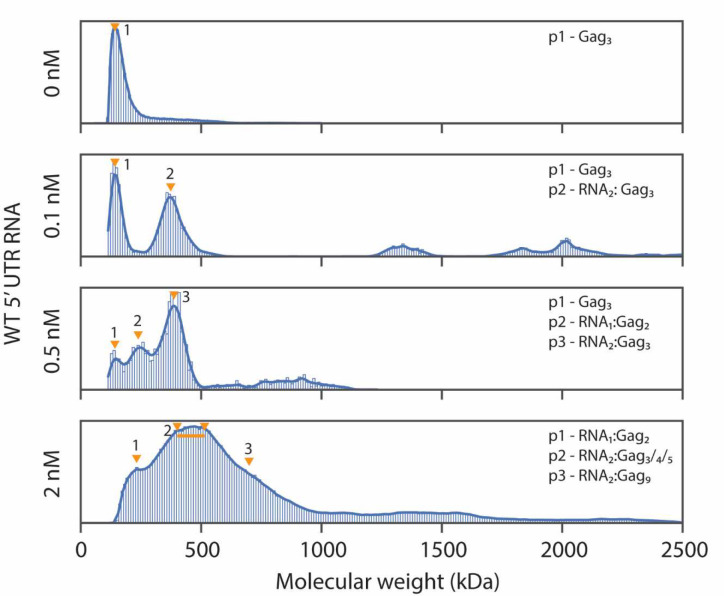
Molecular weight distribution of Gag with 5’ UTR RNA in histogram and kernel density plot. Molecular weight distribution probability density plot (bars) of 50 nM myr-Gag mixed with different concentrations of WT 5’ UTR RNA on SLB. Labeled complex compositions were estimated from kernel density plot peak molecular weights (blue lines). Orange triangles indicate the expected positions of the described complexes, with a short line showing the range a group of complexes are expected to be as individual peaks are not discernable. All sample sizes are larger than 2500 identified particles and collected from at least 3 different recordings with at least 2 different protein preparations.

To investigate lipid-dependent Gag-RNA complexes formation myr-Gag was examined in MSPT experiments on an SLB, alone and with varying amounts of WT 5’ UTR RNA. The results reveal a diverse set of complexes forming in the presence of RNA, with an informative concentration dependence (Fig. 2). In the absence of RNA, a major peak corresponding to a myr-Gag trimer was observed, in contrast to the monomer/dimer species that predominate in solution. This observation strongly suggests that Gag-Gag interactions are enhanced by the presence of lipid, resulting in a predominant trimeric species that is not observed in solution. Substantial Gag-RNA binding is observed at a very low RNA concentration (0.1 nM), with the appearance of a species at ~385 kDa, corresponding to an RNA_2_:Gag_3_ complex (Fig. 2). Small populations of larger complexes also appear, corresponding to higher order oligomeric states of Gag (Fig. 2). However, the myr-Gag trimer peak remains as the dominant species. As the RNA concentration was increased to 2 nM, the myr-Gag trimer peak gradually disappears, and a broad peak representing a mixed species profile of RNA_2_:Gag_3/4/5_ emerges together with other higher-MW complexes (n.b. Gag-RNA complex peak assignment rationale is described in Methods).

These results demonstrate that the presence of even substoichiometric amounts of 5’ UTR RNA enhances Gag oligomerization on the membrane, and that both RNA concentration and the protein-to-RNA ratio affect the final distribution of Gag-complex species on the SLB. In summary, myr-Gag preferentially forms dimers in solution without and with 5’ UTR RNA, but forms trimers or higher multimers when binding to dimeric 5’ UTR RNA in the context of an SLB. This is clear evidence for the cooperative interaction between protein, RNA, and lipid in the earliest steps of capsid assembly. The RNA_2_:Gag_3_ complex appears as the only major Gag-RNA complex when RNA concentration is very low, and it remains a major species across all RNA concentrations tested, making this complex very likely to be the nucleus for assembly.

## Gag dimerization is important for Gag assembly on a SLB

**Fig. 3.**
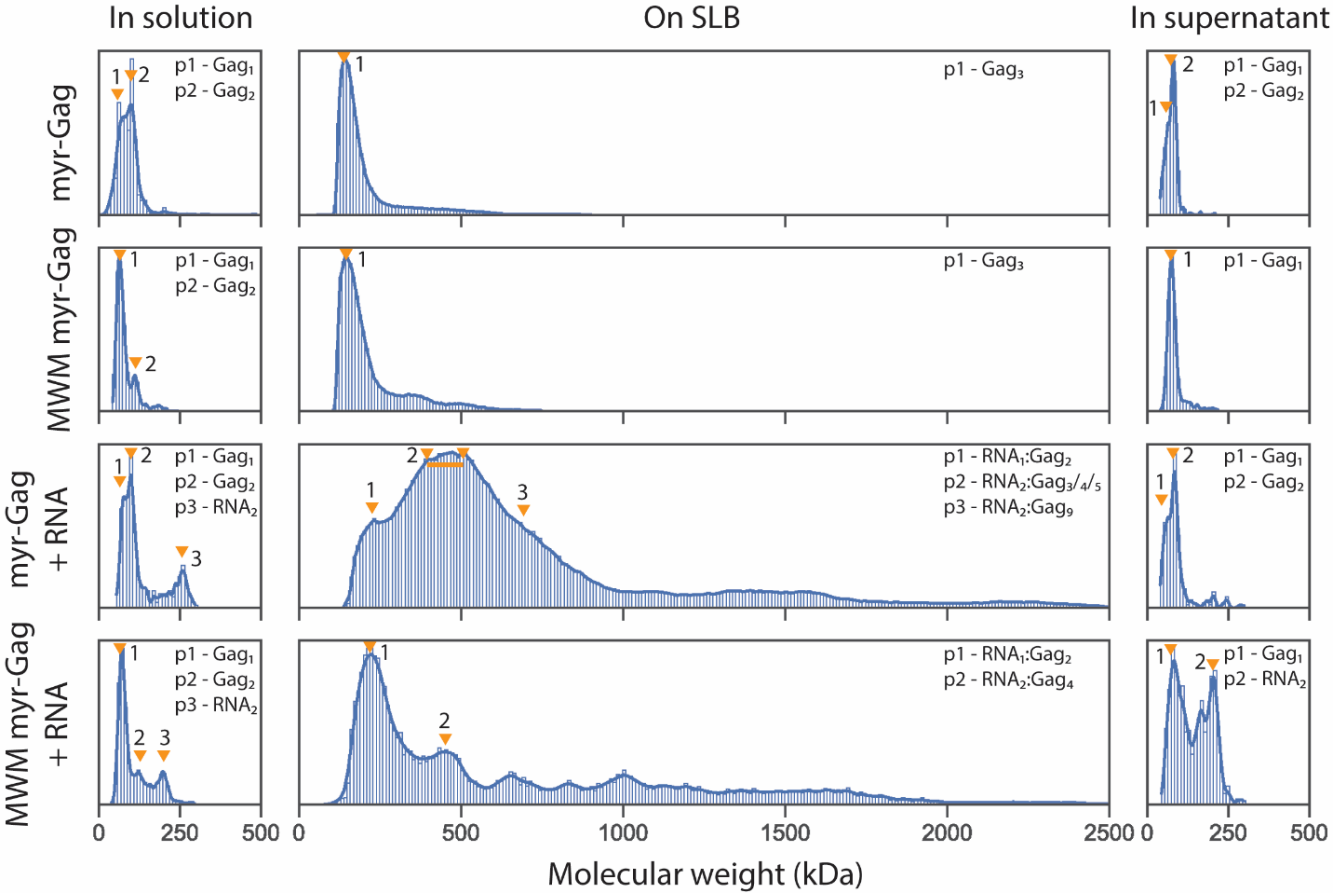
Molecular weight distribution of WT myr-Gag and MWM myr-Gag mutant with 5’ UTR RNA. Molecular weight distribution probability density plot of 50 nM WT myr-Gag or MWM myr-Gag without or with 2 nM 5’ UTR RNA in binding buffer before applying to the SLB (landing assay), on SLB (MSPT assay), or in supernatant solution after SLB incubation (landing assay). Labeled complex compositions were estimated from kernel density plot peak molecular weights. Orange triangles indicate the expected positions of the described complexes, with a short line showing the range a group of complexes are expected to be as individual peaks are not discernable. All sample sizes are larger than 2500 identified particles for MSPT assay data and larger than 400 for landing assay data. Data was collected from at least 3 different recordings with at least 2 different protein preparations.

Gag forms dimers through interactions between the capsid domain (CA), and CA-CA interactions are a key feature of the 2-fold interactions in the Gag immature lattice^10^. To investigate the effect of Gag CA dimerization in the assembly process on the membrane, the MWM myr-Gag mutant (M39A, W184A and M185A disrupting CA-CA dimer interface) was assayed^21^. In solution, MWM myr-Gag forms a smaller proportion of dimers compared to WT myr-Gag, as expected (Fig. 3). In the SLB, MWM myr-Gag also formed Gag_3_ as the predominant species (Fig. 3). Gag is known to form trimers through interactions among the MA domains, and there are also three-fold interactions between the CA domains^22–24^. The formation of Gag trimers in the MWM myr-Gag in the context of SLBs is unaffected by the CA dimerization mutations, and the initial Gag trimer formation does not depend on dimerized Gag in solution.

The mass distribution in the supernatant after SLB incubation was also measured in a landing assay, to determine if the distribution of species in solution was altered by exposure to the SLB. In both cases, there was one broad peak between 55 kDa and 110 kDa, indicating no species larger than a Gag dimer exists in the solution after SLB incubation (Fig. 3).

When RNA is added to the mixture, MWM myr-Gag does not substantially bind to RNA in solution, similar to WT myr-Gag (Fig. 3). However, when applied to the SLB surface, the mutant showed fewer higher order complexes and only a small fraction of Gag_4_:RNA_2_, compared to WT myr-Gag (Fig. 3). The MWM mutation did not abolish the RNA_1_:Gag_2_ complex formation (~220 kDa) that also appeared in WT experiments. Since the MWM mutation impairs Gag dimerization, this result indicates RNA_1_:Gag_2_ can be maintained via both Gag-RNA interactions and Gag MA-MA interactions in the context of the SLB. It is important to note that while MSPT data can discern the molecular weight of complexes, it cannot discern the conformation or geometry of a given complex. Therefore, complexes may or may not be architecturally homogeneous within a given sample, or across samples.

To investigate how a lipid membrane affects the solution species distribution, supernatants from MSPT assays were removed and re-assayed in a solution landing assay. The composition of the supernatant complexes after SLB incubation exhibited distinct distributions for the wildtype and mutant Gag species. The MWM myr-Gag complexes in supernatant exhibited a much larger portion of RNA dimer after 30 min SLB incubation compared to the WT Gag, indicating that MWM myr-Gag cannot recruit 5’ UTR RNA to the membrane surface as efficiently as the WT myr-Gag (Fig. 3).

## Gag assembly in the SLB occurs in small incremental steps

**Fig. 4.**
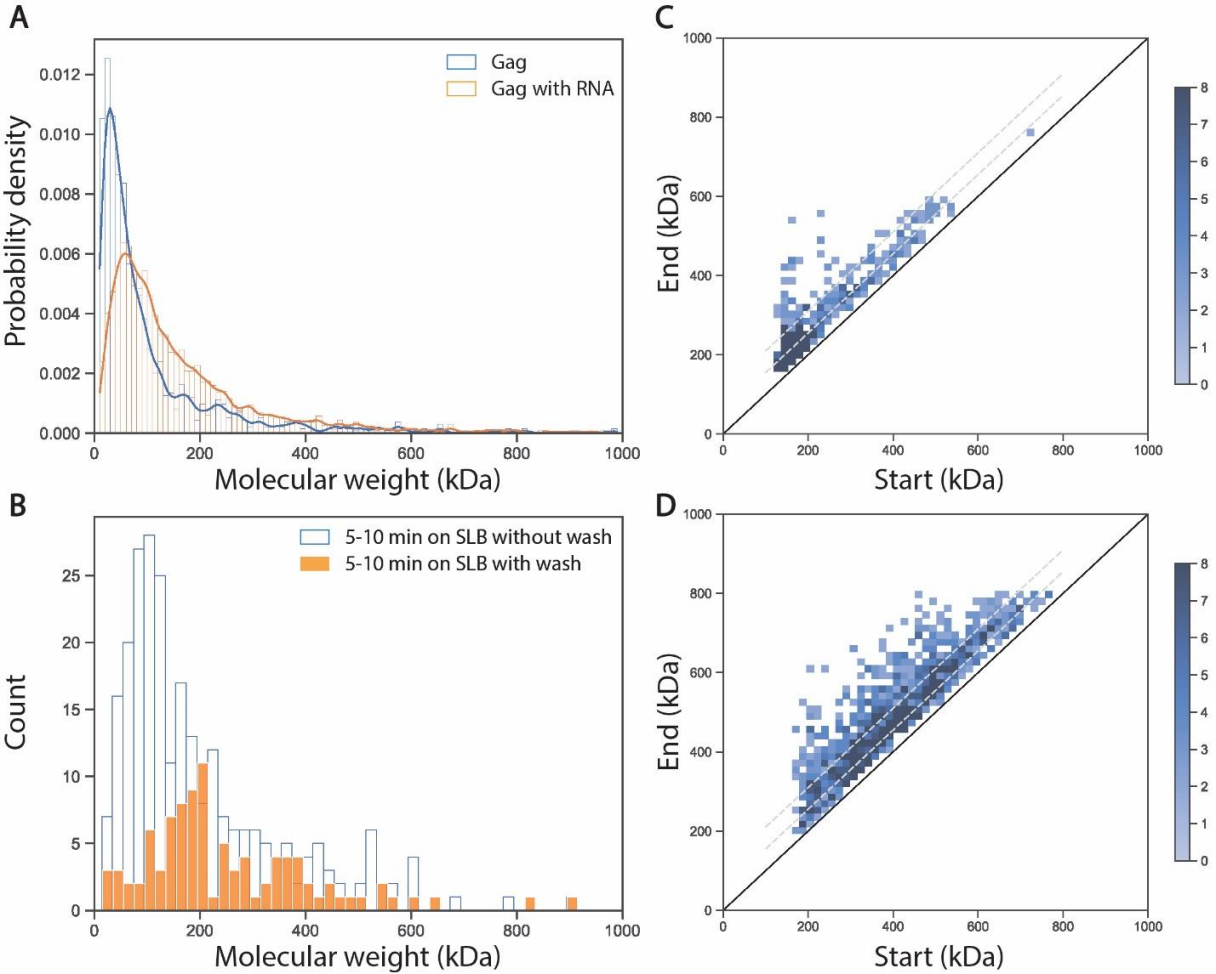
Step size distribution of attachment events and step analysis. (**A**) Probability density of step size distribution for attachment events for 50 nM myr-Gag on SLB (blue) and 50 nM myr-Gag with 2 nM WT 5’ UTR RNA on SLB (orange). (**B**) Histogram of step size distribution for attachment events of 50 nM myr-Gag with WT 5’UTR RNA at 5–10 min after incubation on SLB (blue) and 50 nM myr-Gag with 2 nM 5’ UTR RNA at 5–10 min after incubation on SLB and buffer wash at 5 min (orange). (**C**) Transition density plot of step size distribution for attachment events for 50 nM myr-Gag on SLB, with the two dotted grey line shows the “+ 55 kDa” steps and “+ 110 kDa” steps. (**D**) Transition density plot of step size distribution for attachment events for 50 nM myr-Gag with 2 nM 5’ UTR RNA on SLB, with the two dotted grey line shows the “+ 55 kDa” steps and “+ 110 kDa” steps.

Step detection on time-resolved mass traces was modeled using the Kalafut-Visscher algorithm without manual step-size input^25^, allowing the detection of transitions between different species on the SLB. Comparing the results of myr-Gag alone and myr-Gag with WT 5’ UTR RNA, the step size distributions for attachment events are largely similar, though myr-Gag with WT 5’ UTR RNA samples display some larger steps (Fig. 4A). For both samples the most frequent mass transition is also the smallest mass change, with a molecular weight change of either 1 or 2 myr-Gag (~55 and 110 kDa). At these concentrations, the oligomerization of Gag in the SLB occurs preferentially by adding one or two Gag proteins at a time (Fig. 4A). In addition, the transition density histogram showed that this step distribution is not limited to formation of the very first RNA:protein nucleus, and the addition of 1 or 2 myr-Gags was the major transition observed for subsequent complexes irrespective of their size (Fig. 4C, 4D). Considering the results of the landing assay which showed Gag in solution does not form larger complexes before or after SLB incubation (Fig. 3, Fig. S2D), it is likely that Gag directly adds to the Gag complex cores on the membrane from the solution, instead of from lateral collision on the membrane during the early stages of Gag assembly on the SLB. Indeed, there were no observed events that appeared to correspond to collisions between two or more pre-formed complexes within the SLB resulting in a higher molecular weight species.

Recruitment of Gag from solution provides a mechanism to prevent incorporation of more than 2 copies of RNA into a growing particle by merging of two assembly nuclei that both contain RNA and protein. When RNA is present, the chances of adding two or more Gag’s simultaneously is higher, possibly due to the presence of an RNA scaffold. To test this hypothesis, the supernatant containing Gag and RNA was washed away after 5 min incubation on the SLB, and the step size distribution for attachment events in the next 5 min were recorded and compared to a control group without wash at the same time frame. The result showed that there is a sharp decrease in the most abundant Gag or Gag_2_ steps when the Gag protein reservoir in solution was removed, while RNA_1_:Gag_2_ attachment events persisted, supporting the hypothesis that most attachment events are driven by Gag in solution and not across the lipid membrane (Fig. 4B).

The combination of mass photometry landing and MSPT assays have led to new insights into the early stages of the Gag assembly process. With the data discussed above, we showed the importance of incorporating all three components (myristoylated full-length Gag, 5’ UTR RNA, and the lipid membrane) in the experimental system (Fig. 2). We also characterized Gag complex distributions in solution and on membranes, with Gag monomers/dimers dominant in solution, and Gag trimers dominant on the SLB (Fig. 2, S2). Mutant Gag which disrupts CA domain dimerization showed a defect in forming early Gag complex species and a compromised RNA recruitment ability (Fig. 3). Attachment step analysis also revealed that Gag in solution directly adds to the assembly core on the SLB by steps of one or two Gag proteins at a time, and these steps are due to Gag in solution (Fig. 4).

While a Gag hexamer is the smallest repeating structural unit in the immature Gag lattice, there has been no consensus on the potential role of a Gag hexamer as an assembly intermediate, despite decades of studies^26^. Our data do not support a Gag hexamer being a major assembly step at the concentrations tested, as we did not observe steps containing six Gag proteins, nor did we observe hexameric Gag complexes as abundant intermediates. There has been evidence showing Gag adds to the immature lattice via lower order multimers^27^, and more recently, patterns of an edge expansion model were identified in the immature lattice of a budded virion and on mica surface in AFM experiments^28, 29^, but no similar study has been done on a biologically relevant membrane system focusing on the early assembly steps. Our data support an edge expansion model in early-stage Gag assembly where a Gag hexamer is neither the nucleus for assembly, nor added as a whole to the nucleating complex. Rather, assembly proceeds by addition of Gag monomers and dimers to the edge of an assembly core on a lipid membrane surface where trimerization, likely mediated by MA or CA interactions, plays a crucial role.

**Fig. 5.**
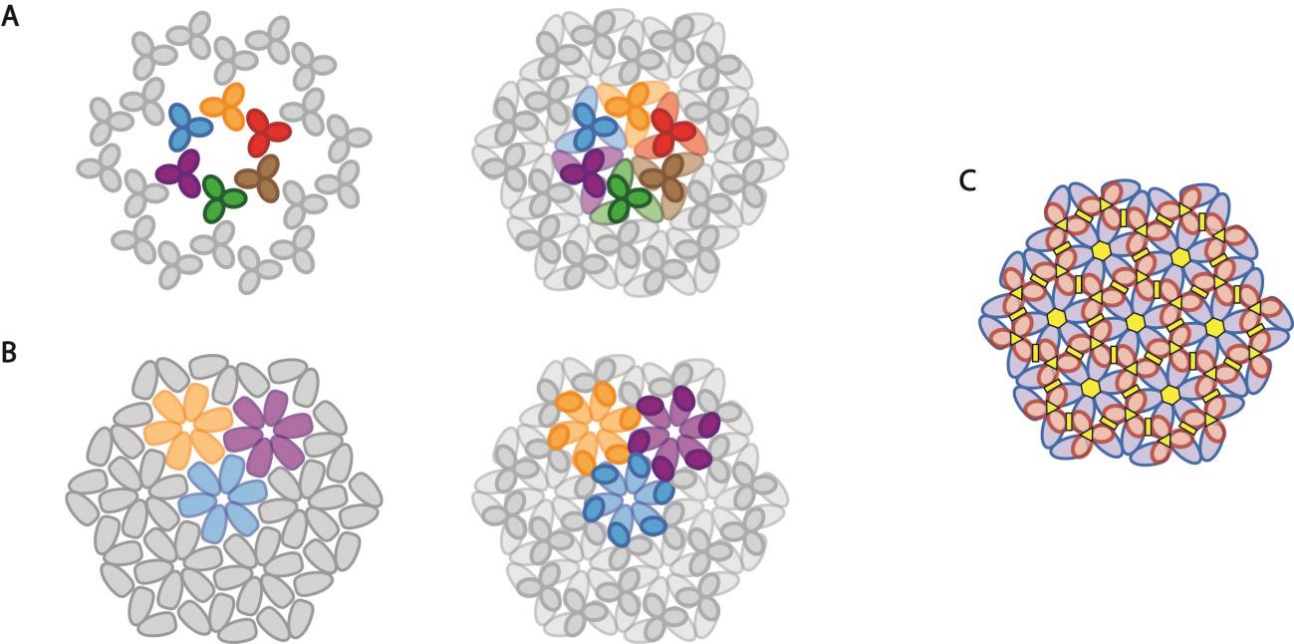
Schematics for Gag lattice. (**A**) Schematic of a trimer of Gag hexamers, with CA-only view on the left and a MA-CA combined view on the right. (**B**) Schematic of a hexamer of Gag trimers, with CA-only view on the left and a MA-CA combined view on the right. (**C**) Schematic of Gag lattice with symmetry axis. Gag MA domain is represented in red and Gag CA domain is represented in blue, with six-fold symmetry axis labeled in yellow.

The interplay between Gag monomers and dimers in solution with Gag trimers in the membrane may reflect the duality of the immature lattice when viewed from the standpoint of the MA and CA domains. Lattice models exist for hexamers-of-trimers for the MA domain (Fig. 5A), and trimers-of-hexamers for the CA domain (Fig. 5B), that have similar spacings and 2-fold, 3-fold, and 6-fold axes. However, cryo-electron tomographic analysis of immature virus particles indicates that while the MA and CA lattices can be independently identified, they do not superimpose directly, presumably due to the flexible linker between the MA and CA domains^23^. Several molecular models have been developed by superposition of the symmetry axes of the MA and CA lattices^11, 13^, which are illustrated schematically in Fig. 5C. There is a key feature of such a model that has important implications for understanding the observed data and resulting assembly model: any given Gag involved in a trimer via MA-MA interactions does not have a common oligomeric partner with any Gag also involved in its hexamer via CA-CA interactions (Fig. 5A). In effect, satisfying the symmetry of both MA and CA lattices during capsid growth may in fact limit the addition of oligomers to the nucleus to one or two at a time.

**Fig. 6.**
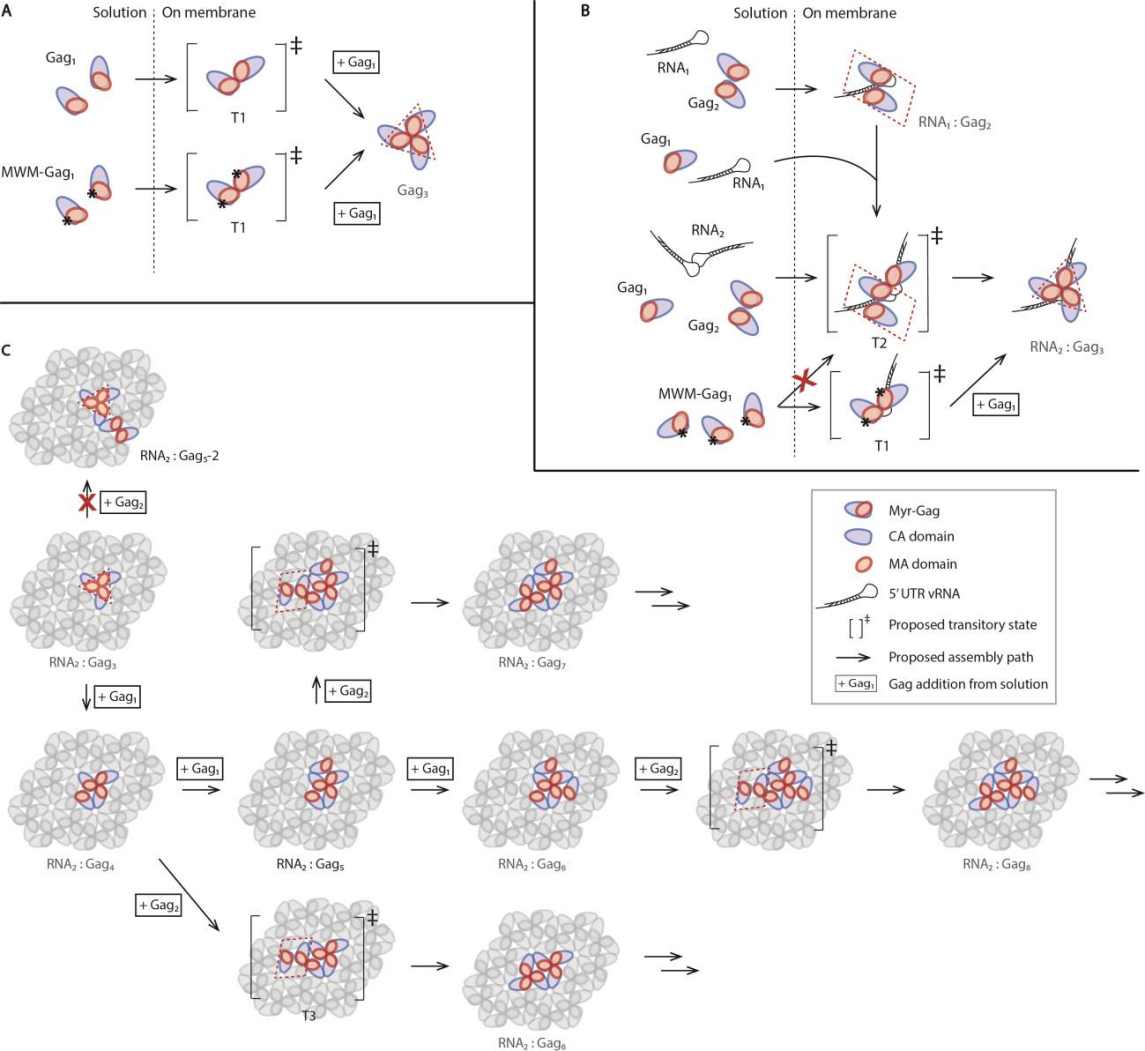
Schematics of proposed assembly pathway for early steps. (**A**) Gag forms a trimer on the membrane in the absence of RNA. Mutations disrupting dimerization do not inhibit trimer formation on the membrane; (**B**) Gag forms a RNA_2_:Gag_3_ complex as the assembly core on the membrane in the presence of RNA. Mutations disrupting Gag dimerization will impede such a process. (**C**) Gag trimer core continues assembly to form complexes containing 4 to 8 Gag proteins. Gag trimer and dimer interaction interfaces restrict further assembly pathway routes. Further Gag assembly can happen with multiple possible alternative pathways in the same pattern and attachment events must be a combination of Gag monomers and dimers to complete all dimer and trimer interfaces in each step. RNA illustrations are omitted in this panel for clarity purpose.

Considering the complex orchestration of dimeric, trimeric, and hexametric interactions between Gag proteins, and based on the observed complex molecular weight distributions and step size distributions for attachment events, a detailed assembly pathway is proposed here (Fig. 6). Since Gag monomer and Gag dimer are the only large populations in solution, we propose that they will dock first on the membrane to form a transition complex T1 when there is no RNA, as mutation disrupting Gag dimerization does not prevent Gag trimerizing on the membrane (Fig. 6A). And when RNA is present, transition complex T2 may also be present in addition to T1, as RNA_2_:Gag_1_ has been detected in both WT and mutant Gag experiments. Both of these transition complexes contain an incomplete Gag trimer interface, and T2 also harbors a completed Gag dimer interface (Fig. 6B). It is not entirely clear how a Gag dimer, presumably dimerized through CA-CA interactions would bind productively through both monomers to a Gag monomer to form the observed trimeric nucleus. We propose a hypothetical transition complex (T2) where one Gag can dock, followed by rearrangement of the interfaces into the predominantly observed Gag_3_ species, driven by the trimer interface of both MA and CA (Fig. 6A, 6B). A key conceptual feature of the dual lattice model tethered by a flexible linker proposed here is that initial interfaces could form by either CA or MA domains with the growing lattice, followed by docking of the tethered partner, followed by rearrangement of the initial docking domain. There is ample evidence for dynamics in the growing lattice with association and dissociation of Gag monomers and dimers, so it is certainly plausible that such rearrangements could occur. In any case, the presumed symmetry of dimeric Gag is likely not present in the observed trimeric Gag nucleus.

This Gag trimer core formation is then followed by addition of a Gag monomer, forming an RNA_2_:Gag_4_ complex with a complete trimer interface and a single dimer interface, satisfying both MA and CA interactions (Fig. 6C). We argue that a Gag dimer addition step onto this Gag trimer core is unlikely because while a partial hexamer can be relatively stable^28^, the fact that Gag prefers trimer over dimer as the smallest complex on an SLB suggests that an exposed dimer interface is more favorable than an incomplete trimer interface, limiting further assembly pathway via RNA_2_:Gag_5_-2 (Fig. 6C). Next, either a Gag monomer can add to the RNA_2_:Gag_4_ complex in the same manner, or a Gag dimer can add to the RNA_2_:Gag_4_ complex to form another transition complex T3, which, similar to T2, would also quickly rearrange to complete another MA trimer interface to form RNA_2_:Gag_6_ (Fig. 6C)

Gag monomers and dimers then add to the complex iteratively in the pattern as exemplified above, eventually forming complete hexamers. When Gag dimerization was compromised by the MWM mutation, the formation of transition complexes is also largely impaired, resulting in fewer Gag_4_ complexes and almost no larger complexes, signifying that adding Gag monomers alone would not sustain the edge expansion. This data supports our model, where while other alternative pathways expanding the complex toward different directions are possible, a combination of Gag monomer attachment events together with dimer attachment events is mandatory to best satisfy 2-fold and 3-fold symmetries in each step.

Our study demonstrated the potential of mass photometry in studies involving protein-protein assembly on a membrane surface. This is the first time that Gag MA domain trimerization has been considered as an important driving force of early-stage Gag assembly on the membrane, instead of Gag CA domain hexamerization. It is also the first study to support an edge expansion model of myristoylated full-length Gag during the nucleation stages of assembly on a lipid membrane. The proximity of the MA domain to the membrane via the myristoyl anchor may explain the observed dominant trimeric species in the assembly nucleus. Once sufficient clustering of Gag occurs by addition of monomers/dimers, the formation of CA hexamers is supported by edge expansion. Remarkably, the dual symmetry nature of the MA and CA lattices imposes a stepwise assembly on the membrane, possibly to ensure smooth formation of an extended lattice by a lengthy series of nearly identical steps. This work integrates many known features of Gag assembly, but provides key insights into the earliest steps of forming the essential lipid-RNA-protein nucleus for RNA packaging and provirion assembly.

## Methods

### Gag expression and purification.

A single plasmid was constructed bearing expression cassettes for both full-length Gag as a C-terminal MBP-(His)6 fusion, and the yeast NMT1 gene, and transformed into *E. coli* DE3 cells. The cell culture was grown at 37 °C until its OD600 reached 0.5 and myristic acid stock solution was added to a final concentration of 50 μM. The cell culture was then incubated at 28 °C for 15 min before adding 1 M IPTG to a final concentration of 0.5 mM. The incubation temperature was kept at 28 °C after induction, and the same amount of myristic acid was added to the culture again after 2.5 h incubation to maintain the myristic acid concentration before collecting the cells after a total 5 h incubation time. The cell pellets were stored at −80 °C before purification.

The myristic acid solution was freshly prepared at 5 mM concentration by first dissolving the myristic acid in 5 mL ethanol and then diluted with 50 mL pre-warmed 0.6 mM BSA at pH 9 (adjusted with NaOH). The solution remained at about 50 °C before use to maintain the solubility of myristic acid.

Cell pellets were resuspended in Gag sonication buffer (pH 7.5, 25 mM HEPES, 100 μM TCEP, 100 μM EDTA, 500 mM NaCl, 20 mM imidazole, 0.1% Tween 20) with 1 tablet of protease inhibitor and incubated on ice for 10 min. The mixture was then sonicated at 4 °C on ice (20 s on, 59 s off, 50% amplitude, 8 min on in total) and was then centrifuged at 10,000 g for 30 min. The supernatant was removed and loaded onto a 5 mL HisTrap column equilibrated with Gag HisTrap A buffer (pH 7.5, 25 mM HEPES, 100 μM TCEP, 100 μM EDTA, 500 mM NaCl, 20 mM imidazole, 0.1% Tween 20, 20 mM imidazole). Gag-MBP was then eluted from the HisTrap column by applying an increasing concentration gradient of Gag HisTrap B buffer (pH 7.5, 25 mM HEPES, 100 μM TCEP, 100 μM EDTA, 500 mM NaCl, 20 mM imidazole, 0.1% Tween 20, 400 mM imidazole) and fractions containing Gag-MBP were pooled together. The pooled fractions were then loaded onto a 5 mL Heparin column (17–0407-01, Cytiva) equilibrated with Gag Heparin A buffer (pH 7.5, 25 mM HEPES, 100 μM TCEP, 100 μM EDTA, 25 μM ZnCl2, 100 mM NaCl), followed by gradient elution with Gag Heparin B buffer (pH 7.5, 25 mM HEPES, 100 μM TCEP, 100 μM EDTA, 25 μM ZnCl2, 1 M NaCl). Fractions containing Gag-MBP were pooled together and concentrated using a 10K concentrator. The final Gag-MBP solution was exchanged into TEV cleavage buffer (pH 7.5, 50 mM Tris, 500 μM EDTA, 1 mM DTT, 500 mM NaCl) and concentrated by a PD-10 desalting column (52–1308-00 BB, GE). TEV (expressed and purified as previously reported) was added to the Gag-MBP solution, and the mixture was incubated at 30 °C for 2 h. The reaction mixture was then loaded onto a Ni-NTA column (30410, QIAGEN) equilibrated with TEV cleavage buffer, followed by washing with 4 mL TEV cleavage buffer. All eluates were collected and concentrated at 15 °C to avoid Gag aggregation, as the solubility tag MBP had been removed. Glycerol was then added to the final Gag solution and the protein was stored at −80 °C in small aliquots. The presence of full-length Gag-MBP and its purity were monitored by SDS-PAGE and the molecular weight of the myr-Gag was confirmed by intact mass spectrometry.

To express Gag without myristoylation, a Gag construct without NMT1 gene was used and myristic acid was not added in the expression culture. All protein expression and purification procedures were the same aside from the protein encoding sequence.

### RNA preparation.

Viral UTR RNA and its variants were made by in vitro transcription (IVT) (AmpliScibeTM T7-FlashTM, ASF3257, Lucigen) using a template containing a T7 promoter made by PCR from a plasmid encoding full-length gRNA sequence. IVT was done at 37 °C overnight, followed by spin-column purification. RNAs were stored at −80 °C in small aliquots. Aliquots of RNA were thawed slowly on ice and then diluted to 1 μM in RNA refolding buffer (pH 7.5, 20 mM Tris, 10 mM NaCl, 140 mM KCl, 1 mM MgCl2). The diluted RNA was refolded by heating it up to 80 °C for 5 min followed by gradually cooling to 10 °C. The refolded RNA was kept on ice before use. The integrity of RNA was verified by urea SDS-PAGE and the conformational change through refolding was demonstrated by native agarose gel electrophoresis.

### SLB preparation.

SLBs were prepared using defined mixtures of phosphatidyl choline (POPC) (850457, Avanti) and PI(4,5)P2 (840046X, Avanti) if needed. The lipids were dissolved in chloroform, mixed in the desired ratios, dried under nitrogen and the residual chloroform was removed in a vacuum desiccator overnight at room temperature. The dried lipid “cake” was then resuspended in SLB buffer (pH 7.5, 20 mM Tris, 10 mM NaCl, 140 mM KCl). The lipid solution was then extruded through a polycarbonate membrane with a pore size of 50 nm using an Avanti mini-extruder (610020, Avanti) for 49 passes to form lipid vesicles with relatively uniform size. The SLBs were prepared by applying the solution of the vesicles to a cleaned glass slide surface immediately after treatment in a plasma cleaner (PDG-32G, Harrick Plasma) at maximum power for 10 min at 0.5 bar air pressure. Unruptured vesicles were removed through extensive washing with SLB buffer after a 15–20 min incubation at room temperature to allow complete membrane formation.

### Mass photometry landing assay.

Glass slides were washed by distilled water, isopropyl ethanol (34863, Sigma-Aldrich), water, isopropyl ethanol, and water again before being dried in an air stream. A gasket (3 mm diameter, 1 mm thickness, GBL103280, Grace Bio-Labs) was carefully placed on a cleaned slide, which was then placed on the mass photometer holder. Gag binding buffer (pH 7.5, 20 mM Tris, 10 mM NaCl, 140 mM KCl, 1 mM MgCl2, 50 μM ZnCl2) filtered by a 0.22 μm filter was added to the well. Sample protein solution or protein-RNA mixture was diluted or mixed in Gag binding buffer and incubated for 5 min before replacing the Gag binding buffer in the well, followed by immediate recording in the mass photometer for 1–2 min at a frame rate of 100 Hz.

### Mass photometry mass-sensitive particle tracking (MSPT) assay.

Glass slides were washed with water, isopropyl ethanol (34863, Sigma-Aldrich), water, isopropyl ethanol, and water again before drying in an air stream. The slide was then treated in a plasma cleaner (PDG-32G, Harrick Plasma) at 0.5 bar air pressure for 10 min at maximum power. A gasket (3 mm diameter, 1 mm thickness, GBL103280, Grace Bio-Labs) was carefully placed on the treated slide, and SLB buffer was immediately applied to all wells. Vesicles were then applied to the well to form SLB. Residual vesicles were washed away with the SLB buffer by gently pipetting in the well without touching the membrane (as complete removal of all vesicles are rather impractical for an SLB containing negatively charged lipid). Gag binding buffer was then introduced into the well. Sample protein solution or protein-RNA mixture was diluted or mixed in Gag binding buffer and incubated for 5 min before replacing the Gag binding buffer in the well, followed by recording of five 5-min movies at a frame rate of 100 Hz after 5 min incubation.

### Mass calibration curves and diffusion control.

The contrast of a set of mass standards was measured for both the landing assay and MSPT on SLBs to convert the scattering contrast into molecular weight.

For landing assay mass standards, 50 nM bovine serum albumin (BSA) or 50 nM alkaline phosphatase was diluted in Gag binding buffer and equilibrated for 5 min at room temperature. The protein standard solution was added to the well of a landing assay set up and the landing events were recorded for 1 min.

For MSPT mass standards, POPC lipid vesicles containing 0.01 mol% biotinylated PE were used to form SLBs. 2.5 nM streptavidin was added to the SLB after the residual vesicles were washed away with Gag binding buffer. Unbound streptavidin was washed away with Gag binding buffer after 10 min incubation at room temperature, followed by the addition of 100 nM biotinylated BSA (A8549, Sigma-Aldrich), 100 nM biotinylated alkaline phosphatase (29339, Thermo Scientific), or 100 nM biotinylated Protein A (29989, Thermo Scientific). The unbound biotinylated protein was washed away after 5 min incubation at room temperature, and movies were recorded for 10 min for each protein.

### Mass photometry data analysis.

For a landing assay, background removal and contrast-to-mass conversion facilitated by standard curve were done using DiscoverMP software with default parameters.

For MSPT experiments, data processing was done using custom Python scripts. The scripts used in background removal and particle identification was a combination of scripts kindly provided by the Kukura lab (University of Oxford) and the Schwille lab (Max Planck Institute of Biochemistry), with specific modifications to meet the current needs.

Background processing was done using a median background subtraction method. To detect particles laterally moving on the surface, this method calculates the pixel-wise temporal median image of a 1001-frame window (half window size n=500) with the frame of interest in the center and uses it as the static background to be removed for that particular movie frame.

$${\text{mobile}\;\text{features}\;\text{frame}}_{i}=\left(\frac{{\text{frame}}_{i}}{\text{median(}\left.{\text{frame}}_{i-n}:\;{\text{frame}}_{i+n}\right)}\right)-1$$

With this median background subtraction process, moving particles on the SLB would no longer appear to be distorted and can be properly fitted by point spread functions (PSFs) to extract contrast for each spot.

To identify spots in the background corrected movies, for each frame a Laplace filter was applied to filter out shot noise, followed by further thresholding to remove small promiscuous spots. A local maximum was identified for each of the candidate spots and its pixel coordinates were noted as the candidate pixel. A 13×13 pixels (84.4 nm per pixel) region of interest was marked for each candidate spot around the candidate pixel in the original image and fitted by the same PSF used in the DiscoverMP software to extract particle contrast and location. Spots at very close separation were rejected at this step to guarantee good PSF fit. Spots with very large contrast (equivalent to more than 5500 kDa) are excluded as their molecular weight far exceed particles in early assembly process and are more likely to be vesicle residues.

After particle identification, trajectories were generated using the TrackPy Python package with minimum length filter set to 20 frames (200 ms), memory set to 1 frame, and maximum allowed step distance set to 5 pixels (422 nm).

For Gag-RNA complexes species assignment, the following considerations were taken into account: 1) as myr-Gag alone would not form complexes larger than Gag3 on SLB, it was assumed that no larger Gag-only complexes would form when RNA was added to the system; 2) under physiological conditions, HIV-1 would only package 2 copies of gRNAs into the newly assembled virions, thus it was assumed here the maximum RNA copy number in each complex species is 2; 3) when combined with mutated monomer gRNA 5’ UTR and incubated on the SLB, the largest complex was RNA_1_:Gag_4_ (data not shown), suggesting 2 RNA molecules need to exist and dimerize for more than 4 Gag proteins to be incorporated into the complex. All peak assignments regarding Gag-RNA complexes were made based on these three premises.

As there are a variety of complexes differing with each other only by ~55 kDa, some species tend to merge into broader peaks in our experiments, making it difficult for multiple gaussian fits to identify the exact molecular weight of each species. To still provide useful and indicative molecular weight information, in this work we plotted the particle molecular weight distribution in both histogram and kernel density plot, and labeled the expected position of each Gag-RNA complexes, instead of the gaussian fit value of the distribution.

### Step detection algorithm and step size analysis.

To detect mass change steps throughout trajectories, a C language implementation of the Kalafut-Visscher algorithm was used to perform denoising and step detection. The algorithm itself does not require any other input except for the time series itself. Previous studies have reported that the length of the time series and the position of potential change points can affect the step detection result and its accuracy. To address this issue, all individual trajectories from a certain group of experiments were concatenated first, and the data was then divided into a series of n segments of equal length using a sliding window of 1000 frames. Each of these individual 1000-frame segments were used as input to the algorithm for analysis, avoiding bias from step positions and the length of the time series. With this sliding window segmentation procedure, step detection was repeated 1000 times shifting the start point by 1 frame in each iteration. After the step detection was done for every segment, the results were pooled together. For a particular time-spot in a trajectory, it had gone through 1000 iterations of step detection cycle, and the frequency of a returning result being a step was used to decide if it is a real change point identified at that particular location with a threshold of 0.75 (if a location returned more than 750 step detected results among the 1000 iteration, it is considered to be an actual step).

The sliding window length and the threshold are related to each other. A larger window length would result in less sensitive step detection, which can be compensated by choosing a smaller threshold. Considering the noise level of the system and the complexity of the Gag oligomerization process, a rather strict threshold was chosen in this project to avoid over-identification of steps.

## Supplementary Material

Supplement 1

## Figures and Tables

**Fig. 1.**
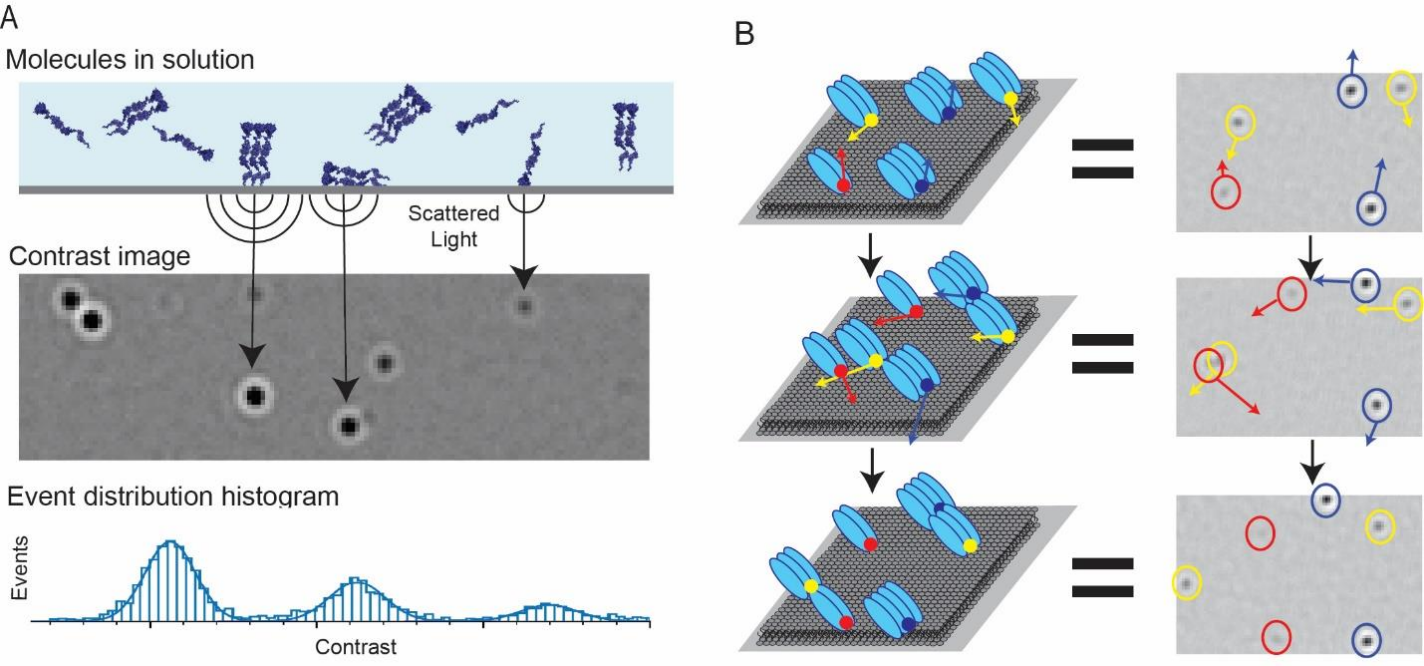
Schematic for mass photometry assays. (**A**) Schematic for mass photometry landing assay, for determination of the distribution of molecular weights in solution. As individual molecules or complexes diffusing in solution absorb nonspecifically to the glass surface, interference of scattered light gives rise to contrast features over time. The contrast is proportional to molecular weight, and a histogram of contrast events gives the distribution of species in solution. (**B**) Schematic for MSPT where oligomeric complexes are diffusing in a 2D membrane. The position and contrast (which can then be converted to molecular weight) are recorded as trajectories over time.
