# Supplementary material for: Early HIV-1 Gag Assembly on Lipid Membrane with vRNA
Source: Res Sq. 2023 Jul 3:rs.3.rs-3060076. Preprint. [Version 1] doi: 10.21203/rs.3.rs-3060076/v1 (PMC10350206; doi:10.21203/rs.3.rs-3060076/v1)
Supplement: Supplement 1 [file NIHPPrs3060076v1-supplement-1.pdf]

Supplementary Materials

Figs. S1 to S2

Movies S1

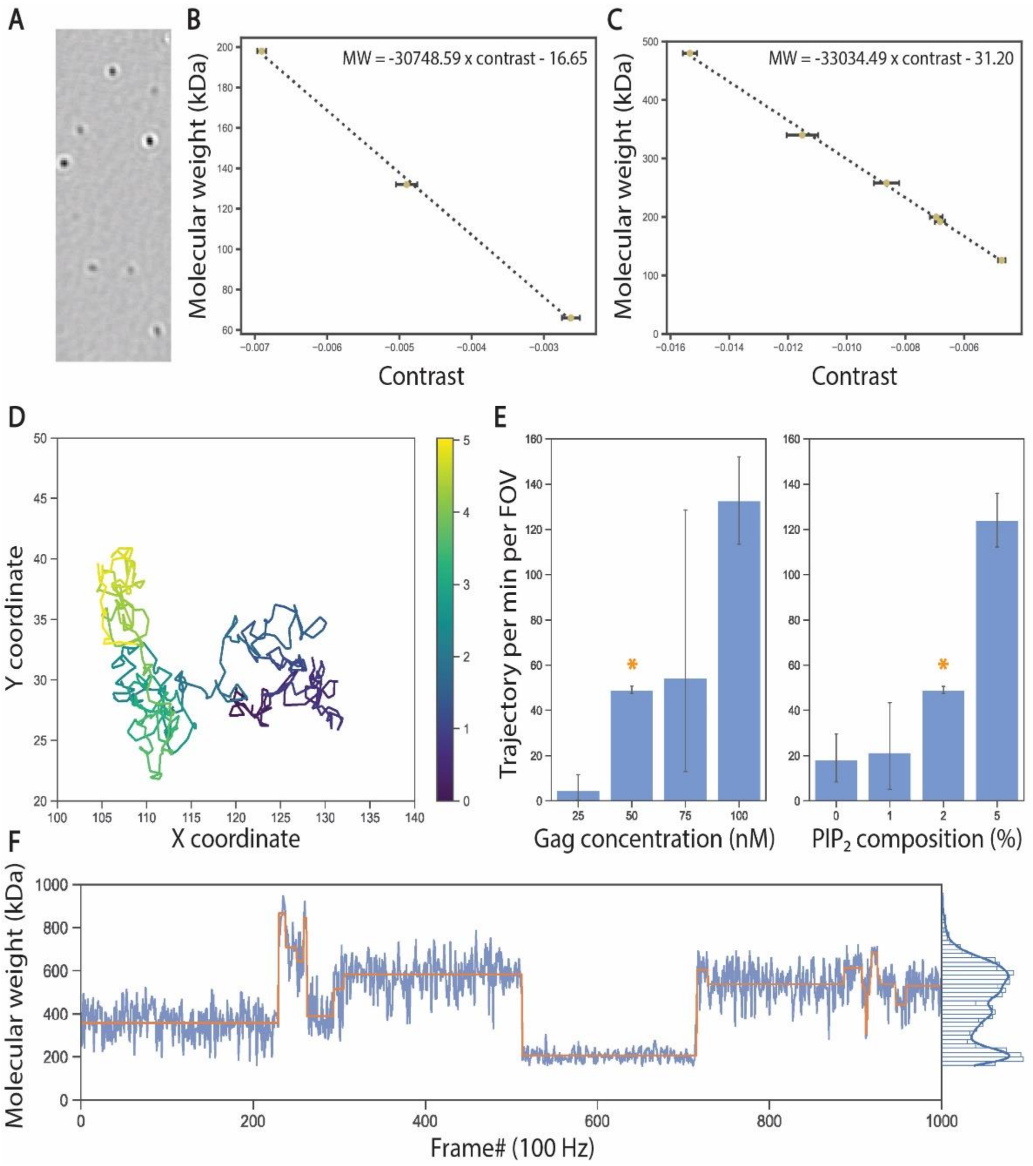

Fig. S1. Samples of mass photometry data, optimization, and calibrations. (**A**) Typical contrast image from the mass photometer in an MSPT assay for myr-Gag diffusing on an SLB (POPC membrane with 2% PI(4,5)P2). The FOV is 9 x 7 microns, composed of 150 x 60 pixels. (**B**) Sample calibration curve for landing assay. BSA was used as a standard protein with its monomer, dimer and trimer peaks being detected. (**C**) Sample calibration curve for MSPT assay. Bio-BSA and bio-AP were used as a standard protein on SLB containing 0.01% bio-PE preincubated with tetravalent streptavidin. (**D**) MSPT measurement of a single trajectory of one single particle over time, consisting of 500 frames collected over 5 s at a frame rate of 100 Hz. The trajectory is colored by time interval. (**E**) Average number of trajectories per FOV as a function of PI(4,5)P2 composition at 50 nM myr-Gag (left), and as a function of myr-Gag concentration at 2% PI(4,5)P2 (right). (**F**) Molecular weight of the particle in each frame over the trajectory, with raw data (blue) and the results of step-detection algorithm (orange) together shown in the plot. The histogram of molecular weight distribution is shown at the right.

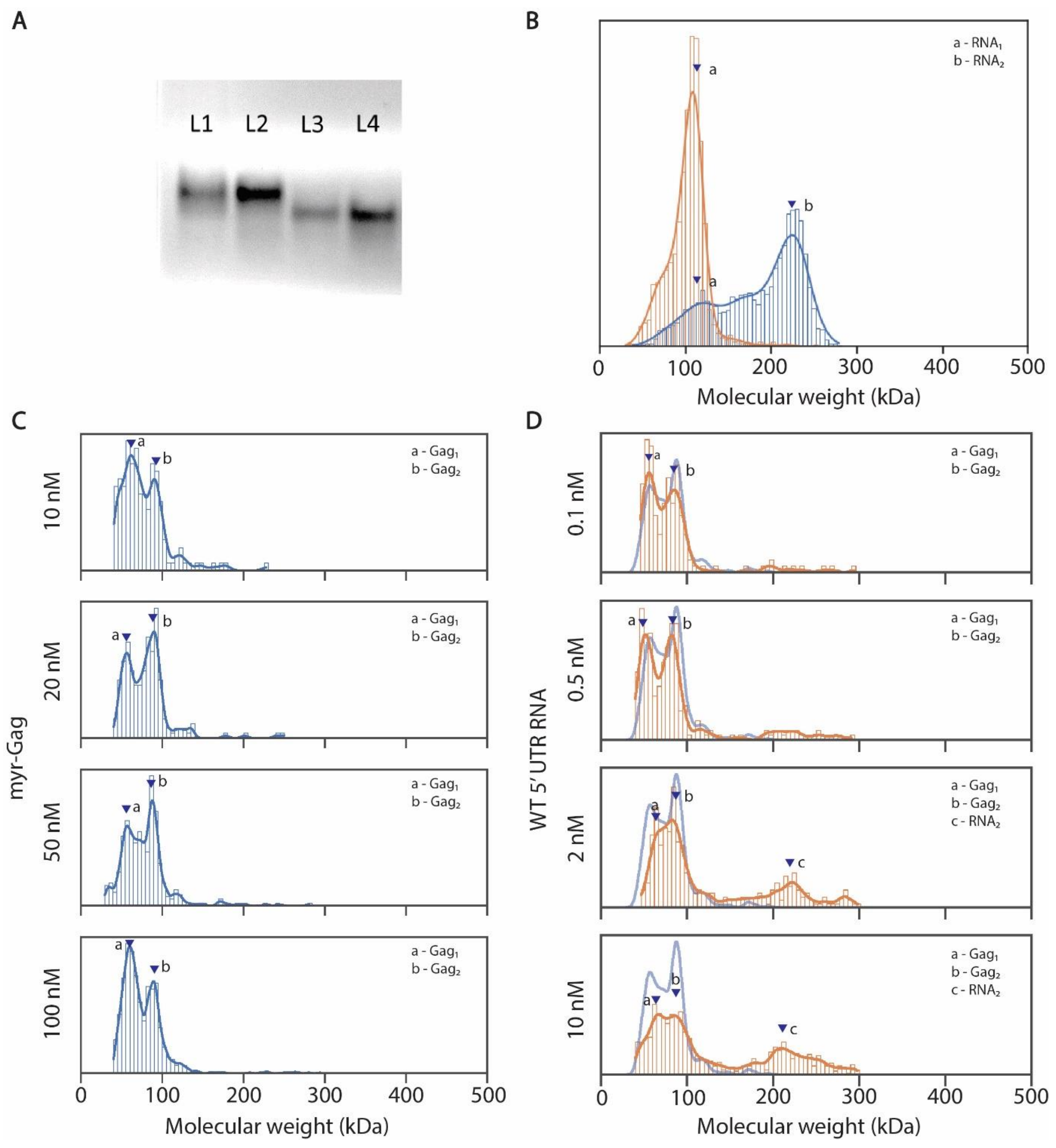

Fig. S2. Molecular weight distribution of RNA and myr-Gag without and with WT 5' UTR RNA. (**A**) Native TB-Mg agarose gel characterization of RNA species. L1: WT 5' UTR RNA; L2: WT 5' UTR RNA, refolded; L3: M2 5' UTR RNA; L4: M2 5' UTR RNA, refolded. (**B**) Molecular weight distribution probability density plot of 50 nM refolded WT 5' UTR RNA (blue) and 50 nM refolded M2 5' UTR RNA (orange), measured by landing assay. (**C**) Molecular weight distribution probability density plot of 10 nM to 100 nM myr-Gag in binding buffer, measured by landing assay. (**D**) Molecular weight distribution probability density plot of 50 nM myr-Gag mixing with different concentrations of WT 5' UTR RNA in binding buffer, measured by landing assay. The light blue line indicates the molecular weight distribution of 50 nM myr-Gag in binding buffer. Labeled complex compositions were estimated from kernel density plot peak molecular weights. Blue triangles indicate the expected positions of the

described complexes. All sample sizes are larger than 400 identified particles and collected from at least 3 different recordings with at least 2 different protein preparations.

Movie S1.

A sample movie of 50 nM myr-Gag and 2 nM RNA on SLB in a mass photometry MSPT assay.
